# Adaptive enhancements of autonomous lane keeping via advanced PER-TD3 framework

**DOI:** 10.3389/frai.2025.1688764

**Published:** 2025-10-17

**Authors:** Xiting Peng, Jinyan Liang, Xiaoyu Zhang, Haibo Yang, Weimin Lei

**Affiliations:** ^1^Shenyang Fengchi Software Co., Ltd, Shenyang, China; ^2^School of Information Science and Engineering, Shenyang University of Technology, Shenyang, China; ^3^School of Computer Science and Engineering, Northeastern University, Shenyang, China; ^4^School of Computer Science and Engineering, College of Arts and Information Engineering, Dalian Polytechnic University, Dalian, China; ^5^School of Artificial Intelligence, Shenyang University of Technology, Shenyang, China

**Keywords:** PER-TD3, sample optimization, lane keeping, autonomous driving, deep reinforcement learning

## Abstract

With the advancement of autonomous driving technology, efficient and safe lane-keeping has become one of the core issues in this field. Currently, Deep Reinforcement Learning (DRL) methods still face challenges such as low training efficiency, slow algorithm convergence, and a tendency to fall into local optima when addressing lane-keeping issues. To address these challenges, a Prioritized Experience Replay (PER) mechanism designed to adapt to the learning process of the Twin Delayed Deep Deterministic Policy Gradient (TD3) is proposed, referred to as PER-TD3, to enhance the learning efficiency and lane-keeping performance of the vehicle in this work. It adjusts the probability of a selected sample by utilizing the difference between the predicted *Q* value and the true *Q* value to assign priority to different samples. By prioritizing samples with higher errors, the algorithm can correct biases in decision-making more quickly, especially when the vehicle deviates from its lane. In addition, introducing a probabilistic sampling mechanism helps to enhance the diversity of samples, ensuring high-frequency playback of high-value experiences, and enabling vehicles to learn accurate and stable lane-keeping strategies in a shorter period. Validation experiments on the TORCS platform demonstrate that the proposed framework can effectively solve the problem of unbalanced training, which is common in DRL, enhances training sample quality, accelerates algorithm convergence, and ultimately improves driving performance while ensuring safety.

## 1 Introduction

With the development of the autonomous driving, enhancing traffic safety and avoiding accidents has become a shared consensus. Among various types of accidents, lane departure incidents have a high proportion, increasing the risks of traffic collisions and rollover accidents during lane changes. Lane-keeping, as one of the fundamental functions of autonomous driving technology, is designed to automatically correct the driving direction and ensure that vehicles remain within their lanes. Researching and achieving this function is a primary condition for the development of autonomous driving technology. In recent years, as advanced driver assistance systems (ADAS) have evolved rapidly (Bisoffi et al., 2017), vehicle lateral control techniques, especially lane departure warning (LDW) and lane keeping assist systems (LKAS), have become a research hotspot, but it is facing challenges including robustness requirements to uncertainties in the traffic environment. Traditional rule-based control methods, such as linear quadratic regulators (Broggi et al., 1999), fuzzy logic (Marino et al., 2011), and model predictive control can provide policy options for autonomous driving vehicles (Zhang et al., 2021). However, these approaches are hardly modeled accurately in complex and variable driving environments.

Recently, research based on deep reinforcement learning (DRL) for lane-keeping tasks has received considerable attention. For example, Peng et al. (2021) proposed an end-to-end lane-keeping framework based on the Dueling Deep Q-Network (DQN), which uses camera images and vehicle motion information as the state space to reduce variance and improve sampling efficiency. While these studies demonstrate the potential of DRL in autonomous vehicle lane keeping, the discussion of this problem and its practical significance can be further expanded. In particular, current DRL methods often face several challenges in lane-keeping scenarios, including sparse reward signals, low sample efficiency, training imbalance, and instability. This training imbalance primarily arises from the insufficient utilization of high-quality data samples, which leads to suboptimal learning efficiency and degraded algorithm performance. Moreover, current mainstream random uniform sampling methods often fail to fully exploit these valuable samples, negatively impacting the real-time decision-making efficiency of autonomous vehicles. Therefore, prioritizing samples becomes crucial, ensuring that high-quality experiences are emphasized during training, which can significantly improve learning efficiency, driving performance, and safety (Yuan et al., 2021).

At present, some researchers have employed prioritized experience replay mechanisms to address issues related to autonomous driving. Specifically, Yuan et al. (2021) proposed a DQN model with a multi-reward architecture (MRA) based on a PER mechanism for highway driving decision-making, which effectively improved driving speed and ensured driving safety. However, both the DQN algorithm and current mainstream lane-keeping methods like Deep Deterministic Policy Gradient (DDPG) suffer from inherent *Q*-value overestimation due to offline learning methods. This occurs because actions selected for updates are based on their potential value rather than real interactions. The TD3 algorithm addresses this by using target networks and minimization operations to reduce *Q*-value overestimation. Compared with the above approaches, combining standard PER with TD3 leverages TD3’s double-critic architecture and target policy smoothing, which allows key samples to be more adaptively utilized during training. This integration results in enhanced sample efficiency, more stable learning dynamics, and superior lane-keeping performance, particularly in scenarios with highly imbalanced training data and continuous action spaces.

To sum up, combining the above discussion, the contribution of this work is as follows:

We propose the PER-TD3 framework for hybrid autonomous lane-keeping, designed to enhance sample quality and driving efficiency while ensuring safety and optimizing overall traffic flow.

Lane-keeping efficiency for autonomous vehicles is optimized by prioritizing samples based on temporal-difference error, leveraging probabilistic sampling for diversity, and refining importance sampling weights to enhance training accuracy.

Experimental results show that the framework surpasses benchmark algorithms like DDPG and TD3 in key autonomous driving metrics, including reward, safe driving distance, and lane-keeping performance such as deflection angle and lateral distance.

## 2 Related work

### 2.1 Rule-based lane keeping

At the beginning, autonomous driving research relied mainly on rule-based strategies in which perception and control were considered as separate modules. For example, Broggi et al. (1999) developed a proportional (P) controller to correct the lateral deviation of the vehicle. To enhance the control effectiveness, a proportional integral derivative (PID) controller will often also be introduced to perform the lateral regulation of the vehicle (Marino et al., 2011). Wu et al. (2008) proposed a lateral controller design that includes full state feedback. Wang et al. (2020) used a sliding mode control strategy to implement the lane keeping function. In addition to these, several other traditional control techniques such as linear quadratic Gaussian (LQG), H infinity (H) control, adaptive control, and fuzzy control are also available. However, the aforementioned classical control methods rely on current and historical feedback signals, and this reliance may lead to slow or insufficiently stable control signal generation. In contrast, model predictive control (MPC) generates optimal control signals based on vehicle dynamics and various types of constraints in a limited time, thus optimizing the overall control effect (Zhang et al., 2021). However, classical control algorithms usually utilize preset parameters and lack the ability to study and adapt to new scenarios. Most of these architectures are based on precise mathematical models, but the actual driving environment is much more complex than these models can describe, and thus these methods may not perform efficiently enough when dealing with changing road conditions.

### 2.2 AI-based lane keeping

Consequently, researchers have been focusing on the application of AI in autonomous driving. Hua et al. (2022) employed the DDPG algorithm to control autonomous driving vehicles, customizing the actor and critic structures in the algorithm specifically for the TORCS environment (Wymann et al., 2000). By evaluating the performance of the algorithm through a number of different driving trajectories, to further validate its effectiveness. Peng et al. (2023) combines the techniques of transfer learning and deep reinforcement learning to conduct innovative research on the challenges encountered in the lane keeping task, especially the low sample efficiency and high time cost. Eventually, the learning speed of the algorithm is accelerated and the efficiency and performance of the overall framework is improved. Zhou et al. (2023) combines a robust x-aware network with transfer learning and fine-tuning techniques to propose an advanced lane keeping assistance system designed for autonomous driving vehicles to accurately predict steering angles. By analyzing photographic images, the model effectively learns human driving knowledge and provides an accurate estimate of the steering angle required to safely maintain the lane.

More recently, some researchers have been considering training directly in the real world. Hong et al. (2024) applied the DDPG algorithm for the first time to a fully autonomous driving vehicle operating in a real-world environment. By randomly initializing the model parameters, which enabled the system to perform lane-keeping tasks in very few driving instances, by simply utilizing monocular camera images as inputs, to learn and master a strategy for performing the lane-keeping task. In addition to this, existing research is focused on understanding and predicting driver behavior and decision-making processes by focusing on driver intent. The system proposed by Wei et al. (2024) integrates adaptive driver characteristics to align with individual driving habits and intentions. A new lane departure decision model is proposed that utilizes temporal and spatial domain fusion to efficiently identify the driver’s intent to change lanes, thereby informing the system decision (Yin et al., 2020). Kendall et al. (2019) neural adaptive control based Lane Keeping Assist System (LKAS). The proposed control strategy synergizes a non-deterministic adaptive control design scheme, adaptive radial basis function based neural network (RBFNN), to capture the human driver’s lane keeping steering behavior.

However, these cutting-edge scientific efforts have also encountered a common challenge: the efficiency of data samples. The development of autonomous driving systems relies on a large number of high-quality data samples for training and optimization, but the reality is that collecting these data is both expensive and complex. Especially in autonomous driving technology, the high cost of acquiring real driving data must be coupled with the high quality and diversity of the data in order to develop models that can be widely adapted. This requires researchers to not only interact with the environment on a large scale to collect data, but also to manually filter and process the data to ensure that the training uses high-quality samples. Only in this way can the training efficiency and performance of driving strategies be effectively improved, thus ensuring the safety and reliability of autonomous driving.

## 3 Materials and methods

### 3.1 Markov decision process (MDP)

Reinforcement Learning (RL) tasks are usually described using MDP. The specific details are described as follows.

*State space*: The state information of the network input is derived from the observation of the vehicle and its surroundings by the TORCS environment used, which contains the following aspects, such as acceleration, brake, clutch, gear, steering wheel and other information, and the input state is set to be continuous. As shown in Table 1.

**Table 1:** Partial status information.

Parameter	Configuration
Angle	[−*π*, *π*]
Speed*X*	(−*∞*, +*∞*) (km/h)
Speed*Y*	(−*∞*, +*∞*) (km/h)
Speed*Z*	(−*∞*, +*∞*) (km/h)
Track	(0, 200)
Rpm	[0, +*∞*] (rpm)
WheelSpinVel	[0, +*∞*] (rad/s)
Damage	[0, +*∞*]
DistFromStart	[0, +*∞*] (m)
DistRaced	[0, +*∞*] (m)
Focus	[0, 200] (m)
Fuel	[0, +*∞*] (L)
Gear	[−1, 0, 1, 2, 3, 4, 5, 6]
*Z*	(−*∞*, +*∞*) (m)

*Action space*: The output action space is continuous, with the steering wheel ranging from −1 to 1. Additionally, the action space includes throttle acceleration, where 0 means no acceleration and 1 means maximum acceleration, as well as the braking status. As shown in Table 2.

**Table 2:** The information of action.

Parameter	Configuration
Steering of the vehicle	[−1, 1]
Throttle of the vehicle	[0, 1]
Brake	[0, 1]

*Reward function*: The reward function is designed by considering the following aspects. First, collisions are still the primary concern, as they are one of the most critical events to avoid during task execution. A negative reward is given when a collision occurs. Second, for the lane-keeping task, it is important to ensure that the vehicle remains within the lane and does not cross the yellow lines on either side of the road. A negative reward is given if the vehicle goes beyond the designated lane. Finally, the rewards during the vehicle’s movement are considered: a positive reward is given for any positional movement of the vehicle, while a penalty is applied if the vehicle is detected to be stationary. The specific formulations of positive and negative rewards are defined in Equations 1–4:


(1) $$R_{\text{damage}}=-2$$

(2) $$R_{\text{outlane}}=-200$$

(3) $$R_{\text{static}}=-2$$

(4) $$R_{\text{forward}}=5$$

This reward is calculated at each step and then a summing operation is performed in each episode to get the final reward value, as shown in Equation 5.


(5) $$R_{\text{total}}=R_{\text{damage}}+R_{\text{outline}}+R_{\text{static}}+R_{\text{forward}}$$

### 3.2 The proposed framework: PER-TD3

**Figure 1:**
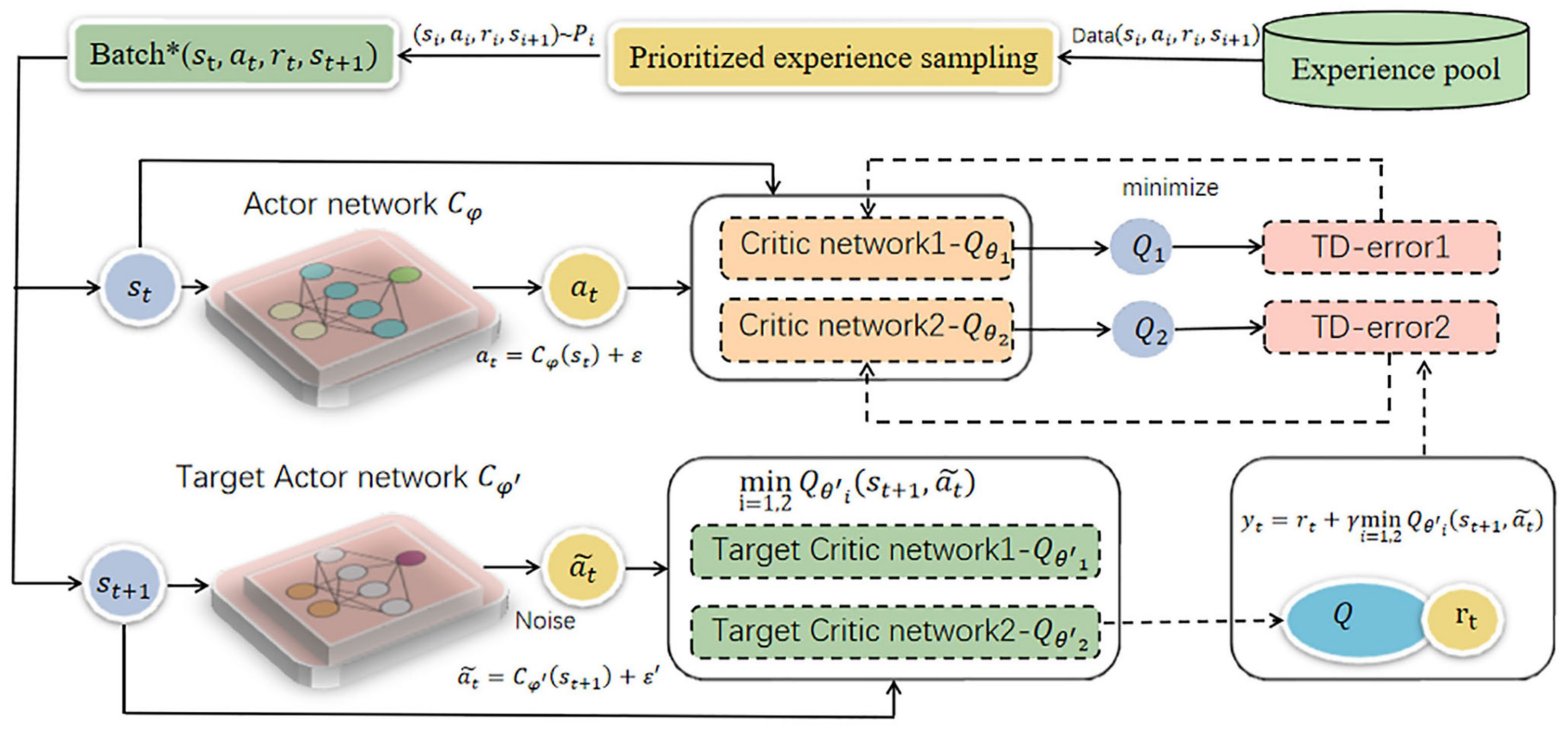
The structure of PER-TD3.

The overall framework is shown in Figure 1. Initially, data samples in the form of quadruples (*s_t_*, *a_t_*, *r_t_*, *s*_*t* + 1_) are generated by the ego vehicle and environment interaction and stored in the experience pool. As interactions continue, the experience pool accumulates more samples of varying importance levels, the important samples are filtered and sent to the network for training. Preferential experience sampling, based on TD-error, prioritizes samples with larger discrepancies between predicted and true *Q*-values. It is defined as shown in Equation 6:


(6) $$\delta_{t}=r_{t+1}+\mathrm{\gamma Q}(s_{t+1})-Q(s_{t})$$

where *r*_*t* + 1_+ γQ(*s*_*t* + 1_) is the true value and *Q*(*s_t_*) is the estimated value. However, this mechanism prioritizes the samples with the maximum TD error, which improves the training efficiency but may reduce the diversity of samples and introduce the risk of network overfitting. To overcome the aforementioned challenges, we introduce probabilistic sampling, an approach maintains a positive relationship between sample priority and TD-error. The probability of sampling each sample can be defined as shown in Equation 7:


(7) $$P(i)=p_{i}^{\alpha }|\sum_{k}p_{k}^{\alpha }$$

where the role of *α* is to adjust the degree of prioritization, then $p_{i}^{\alpha }$ indicates the priority of data sample *i*. When the value of *α* is 0, it corresponds to uniform sampling, as shown in Equation 8:


(8) $$P(i)=\frac{1}{k}$$

this sampling approach ensures all samples are replayed by avoiding neglect of those with low TD-error. There are two ways of defining for $p_{i}^{\alpha }$, namely proportional prioritization and ranking-based prioritization. The former defines the priority of the samples as shown in Equation 9:


(9) $$P(i)=\delta_{i}+\varepsilon$$

where $\delta_{i}$ denotes the error, *ε* is a tiny positive number, is used to ensure that each sample can be sampled once. The ranking-based approach defines the sample priority as shown in Equation 10:


(10) $$P(i)=\frac{1}{\mathrm{rank}(i)}$$

where *rank*(*i*) is the ranking of the *i*th sample after sorting the samples based on TD-error. It uses an indirect ranking method, dividing probability intervals based on rankings and uniformly sampling from each interval. In this study, however, we adopt the proportional prioritization method (Proportional Prioritization) instead of the ranking-based approach, as preliminary tests showed that it provides more stable convergence for lane-keeping tasks in continuous action spaces. The above methods improve the quality of the samples sent to network training, but at the same time, may lead to a biased expectation of the *Q*-value. Therefore, importance sampling weights are introduced as shown in Equation 11:


(11) $$\omega_{i}={\left(\frac{1}{N},\frac{1}{P(i)}\right)}^{\beta }$$

this weight is employed to balance unbiased and high utilization. If the value of *β* is 1, it means that the nonuniform probability is fully compensated. With stability in view, the max*_i_ω_i_* are utilized to normalize the weights so that there is only downward scaling of what they are updating.

Enter the above sample into the network to complete the next training. During the update phase, the Actor target network and the two Critic target networks employ a soft update approach to iteratively adjust network parameters. It is expressed as shown in Equation 12:


(12) $$\theta_{i}^{\prime }\leftarrow \tau \theta_{i}+(1-\tau )\theta_{i}^{\prime }$$

where *i* takes the value of 1 or 2 and τ is the update factor to be satisfied much less than 1. The Critic network updates parameters iteratively by minimizing the loss function, which is computed using the target *Q*-value and predicted *Q*-value as shown in Equation 13:


(13) $$L(\theta_{i})=E[{\left(v_{t}-Q(s_{t},a_{t}|\theta_{i})\right)}^{2}]$$

where *y*(*t*) represents the target *Q*-value, $Q(s_{t},a_{t}|\theta_{i})$ is the output obtained from two Critic networks optimized by the adaptive learning rate. The Actor network parameters φ is updated based on the *Q*-value gradient completion of the Critic networks. Its loss gradient can be defined as shown in Equation 14:


(14) $$\nabla J(\phi )=\frac{1}{n}\sum_{j=1}^{n}(\frac{{\nabla_{a}Q(s,a|\theta_{i})|}_{s=s_{i},a=\mu (s_{i})}}{{\nabla_{\phi }\mu \prime (s|\phi )|}_{s=s_{i}}})$$

where $\nabla_{a}Q(s,|a,|\theta_{i})$ is the gradient of the *Q*-value of the Critic network. $\nabla_{\phi }\mu \prime (s|\phi )$ is the gradient of the Actor network. To enhance method robustness, noise is added to the Actor target network. ε represents random noise added to ensure comprehensive data training during the process, as shown in Equation 15:


(15) $$\mu \prime (s_{t})=\mu (s_{t}|\phi_{t})+\varepsilon$$

Combining with Equation 3, the sampling weights are shown below, where β is a hyperparameter used to smooth out high variance weights and moderate the influence of prior experience playback on results. The loss function of its Critic network is shown in Equation 16. The above algorithm is detailed in Algorithm 1.


(16) $$L(\theta_{i})=E[\omega_{j}{\left(r_{t}+\gamma \min_{i,1:2}Q(s_{t+1},|{\tilde{a}}_{t},|\theta_{i}^{\cdot })-Q(s_{t},|a_{t},|\theta_{i})\right)}^{2}]$$

**Algorithm 1:**
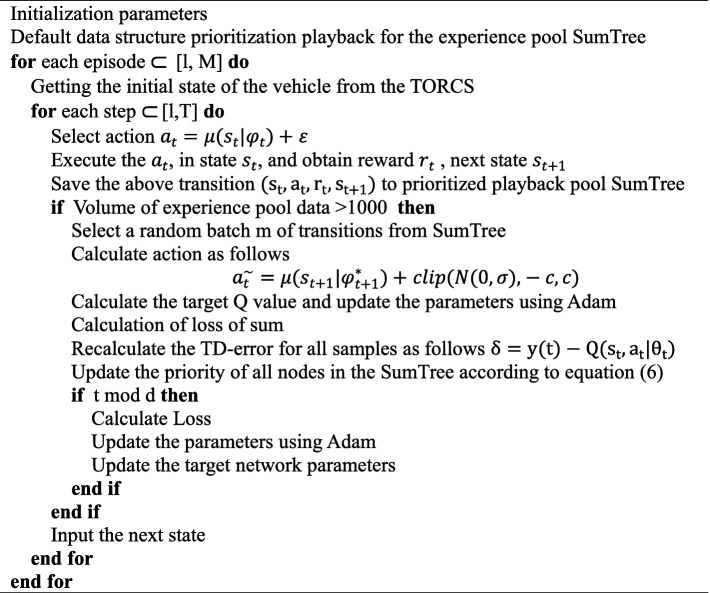
Framework of PER-TD3.

## 4 Results

### 4.1 Simulation settings

**Figure 2:**
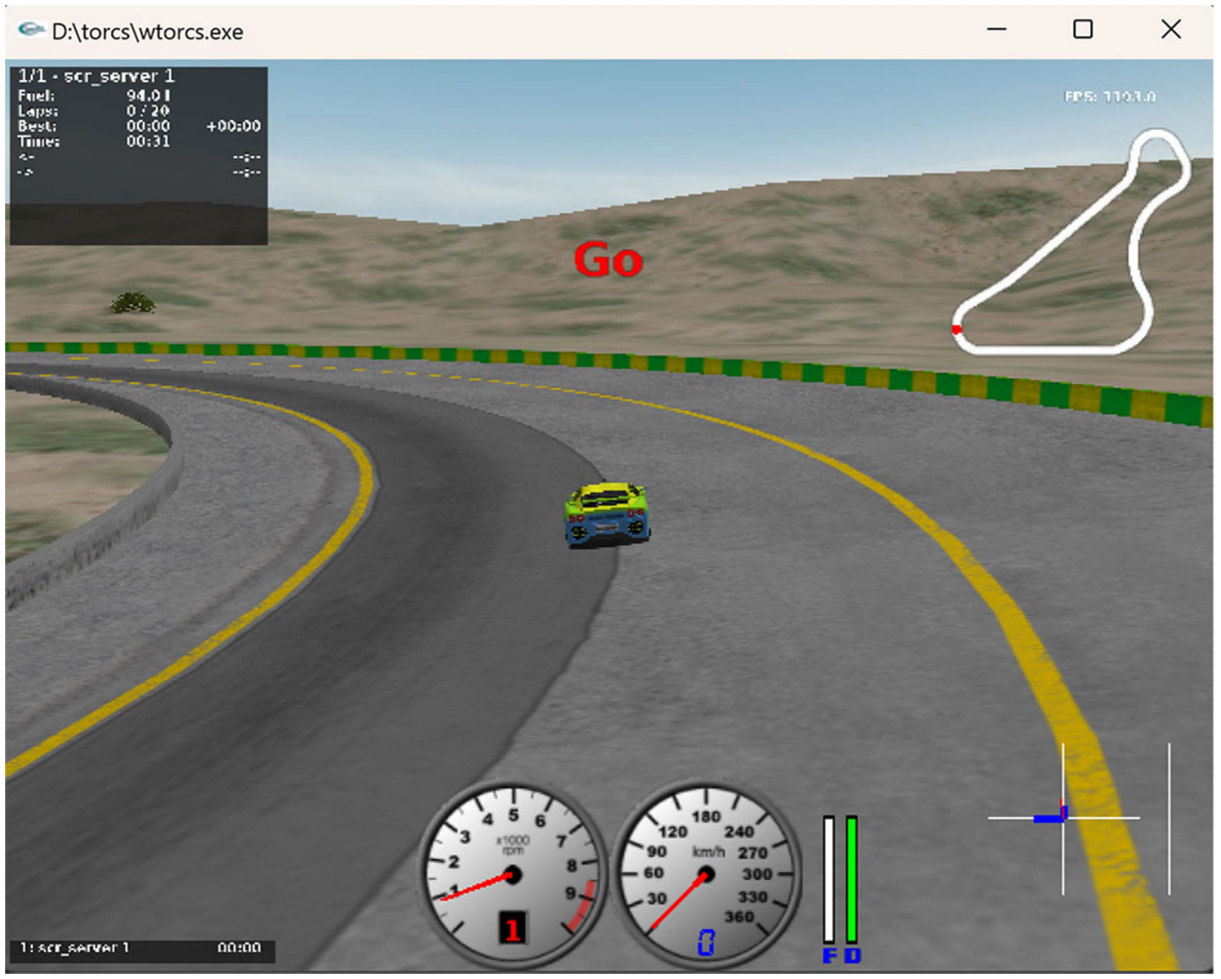
Torcs interface.

In order to realize the real-time interaction between vehicles and lanes, TORCS is selected as the simulation environment for this problem (Wymann et al., 2000). We choose CG Speedway number 1 which is relatively closer to the real track. As shown in Figure 2. The experiment was done on i7-11700 k CPU device with 32 GB of RAM. As shown in Tables 3, 4.

**Table 3:** The track parameters of CG speedway number 1.

Parameter	Configuration
Track length	2,057.56 m
Track width	15 m
Pothole	20

**Table 4:** The description of experimental parameters.

Parameter	Configuration
Minimum batch size	128
Discount factor	0.99
Updating factor *T*	0.01
Experience playback pool capacity	10^6^
Actor network learning rate	0.001
Critic network learning rate	0.002
Delayed update	3

### 4.2 Experimental evaluation indicators

*Reward*: Since the framework as a whole is still designed based on reinforcement learning, its core is still the interaction between the agent and the environment, which guides the next action through the reward value. Therefore, the reward value remains the most critical evaluation indicator in our study, which represents the level at which our trained agent perform the autonomous driving lane keeping task.

*Safe driving distance*: In conjunction with the design of our algorithm, the current turn of the agent vehicle is terminated if a collision occurs during training, and the setting of the safe driving distance represents the normal collision-free forward movement of the vehicle. Therefore, this indicator and the performance of the algorithm, as well as the safety of the vehicle to perform lane keeping, constitute a positive correlation, which is also one of the key indicators reflecting the performance of the algorithm.

*Angle of divergence*: Since we are validating our designed PER-TD3 algorithm based on a lane keeping task, we want the agent vehicle to stay in the middle of the road as much as possible to ensure driving safety. Therefore, the closer the deflection angle is to 0, the better the algorithm performance is represented.

*Distance between vehicles and yellow lines at each end of the road*: Referring to the design of evaluation indicators by other researchers in the field, we introduced the distance between the vehicle and the yellow line at each end of the road to assess the effectiveness of lane keeping enforcement. The distance between the vehicle and the yellow line on the left side was set as positive, and the distance between the vehicle and the yellow line on the right side was set as negative. This indicator takes the absolute value of both sides to make the difference, and the smaller the result, the better the performance of the algorithm.

### 4.3 Experimental effect analysis

1. Analyzing driving effects based on autonomous driving

**Figure 3:**
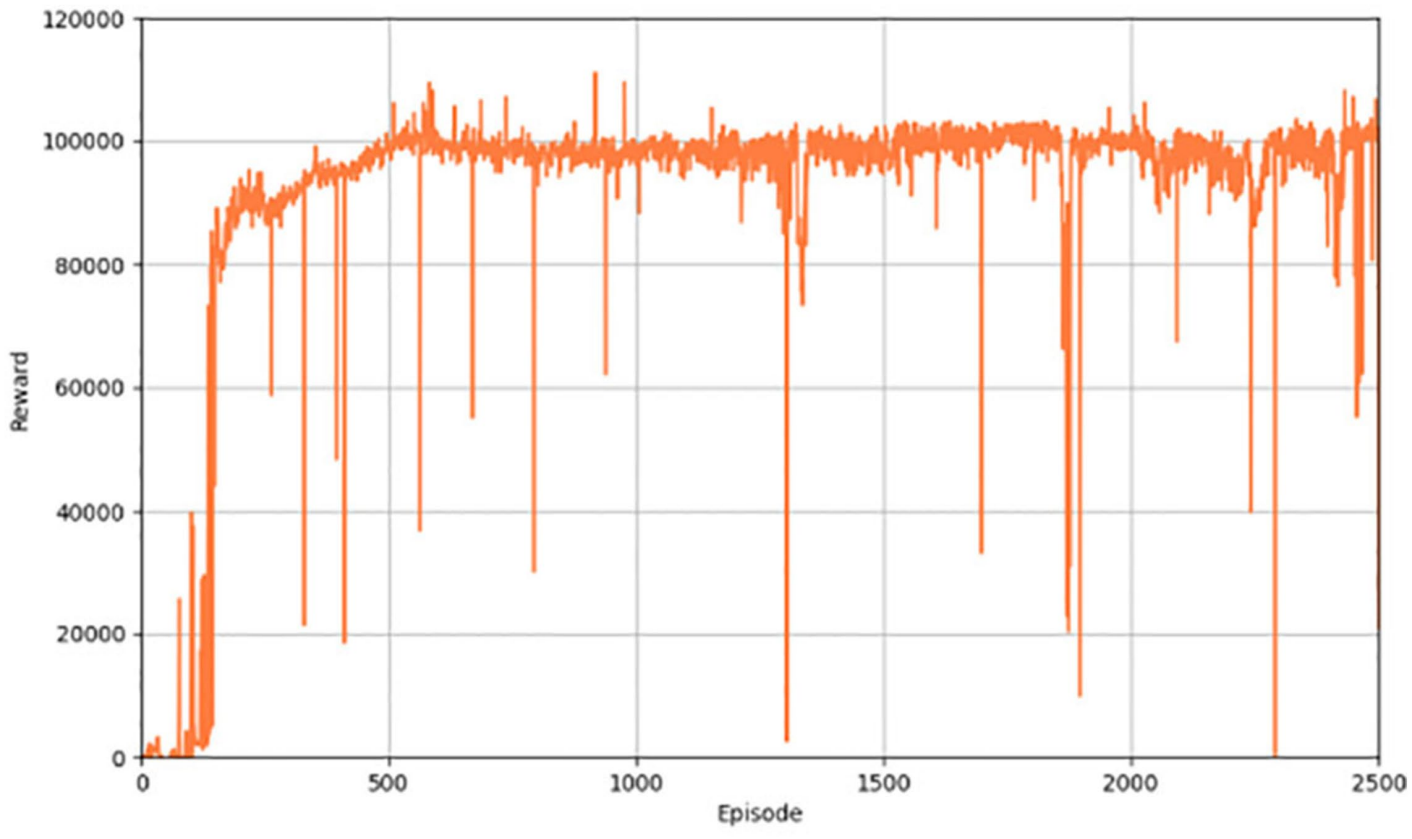
Reward values for the PER-TD3.

**Figure 4:**
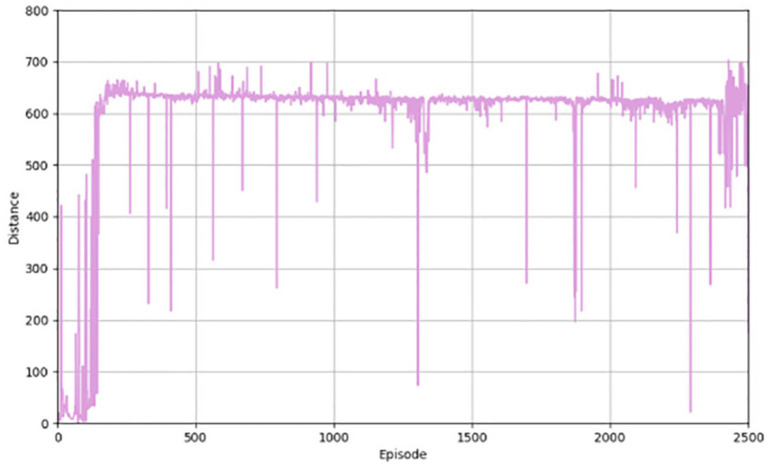
Safe driving distance for the PER-TD3.

Figure 3 shows the performance of the algorithm in terms of reward value. When the training starts pre-training, the results are not very good, but as the training of the network continues, the ability of the network to generate actions continues to improve, and the corresponding reward value continues to increase. Figure 4 illustrates the distance a vehicle can safely travel. It reflects the maximum distance at which the intelligent body vehicle performing the lane keeping task can safely travel without collision in each round. Similar to the overall trend of the reward value, the distance value also increases gradually with the increasing number of training rounds to reach a decent level and converge.

2. Analysis based on the performance of specific lane keeping tasks

**Figure 5:**
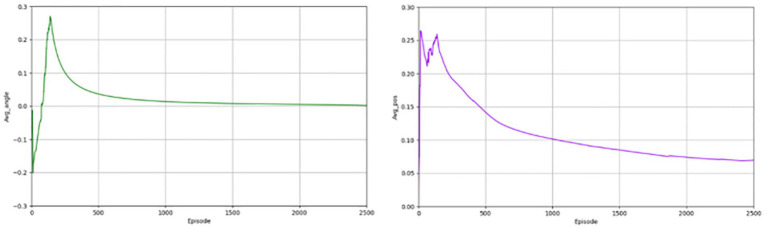
Angle of deviation and lateral distance for the PER-TD3.

Figure 5 depicts the degree of deviation of the autonomous driving vehicle from the road during the forward progress and the gradual equalization of the distance of vehicles from the two ends of the road, indicating a gradual improvement in safety. In the pre-training period, the value fluctuates around 0, indicating that lane deviation occurs from time to time, and along with the continuous training of the network, the deviation becomes less.

**Figure 6:**
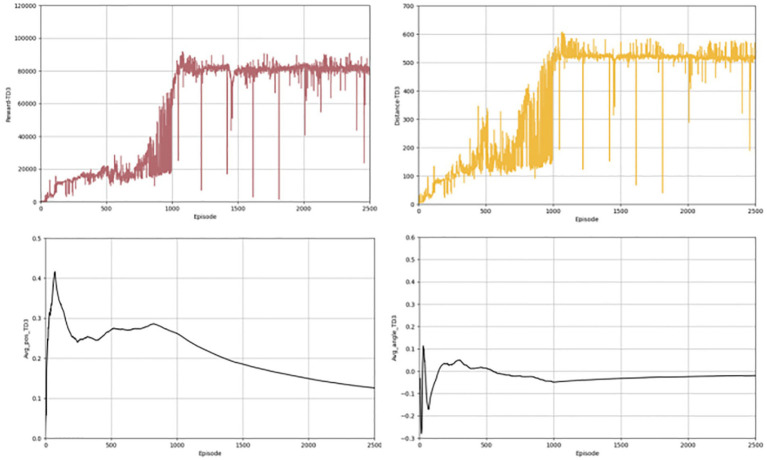
Results for TD3 indicators.

**Figure 7:**
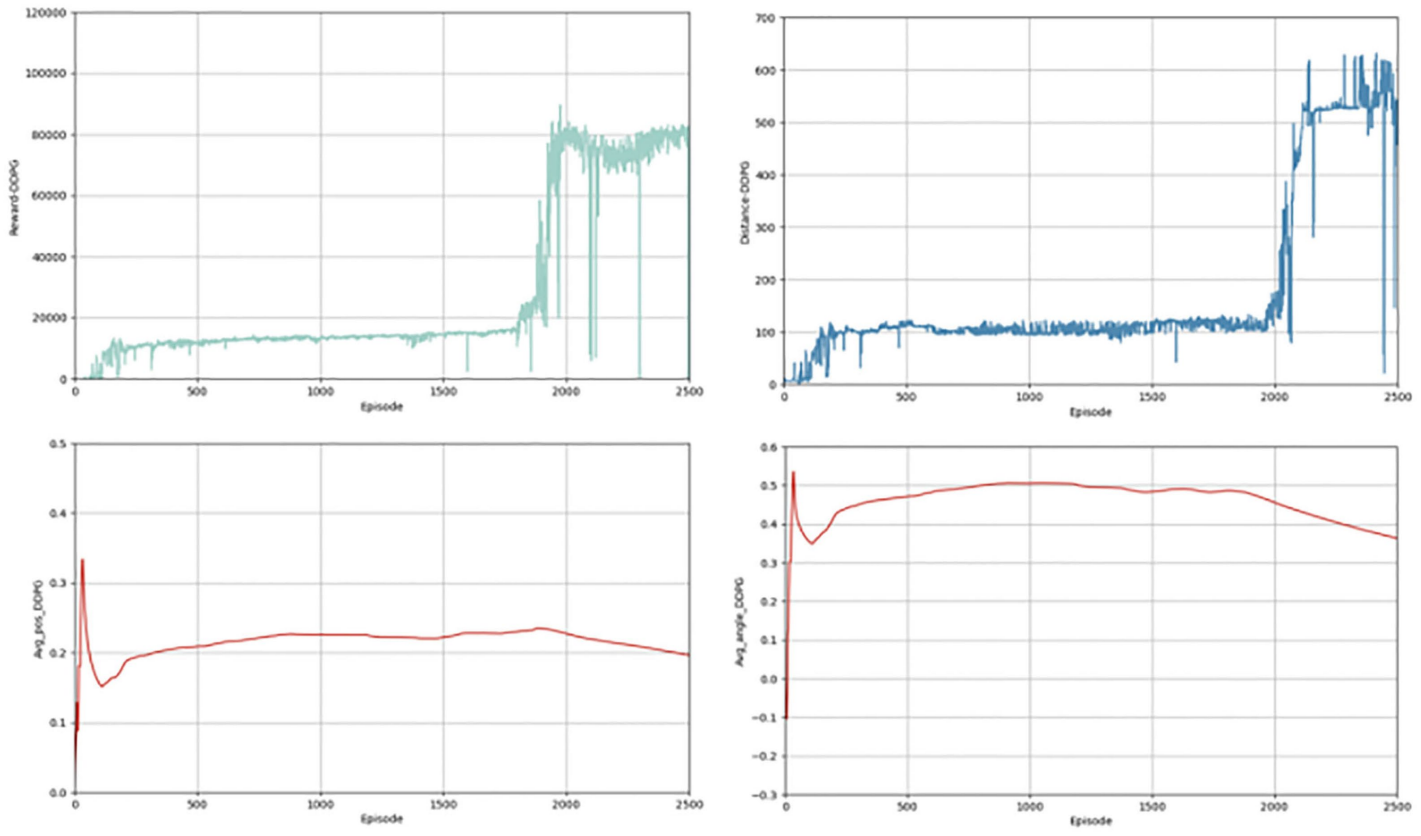
Results for DDPG indicators.

In the above section, we give the evaluation criteria from different aspects of the autonomous driving lane keeping problem, establish a complete evaluation system, and show the experimental results of the algorithms. Next, we present a comparison with the effects of other algorithms, chosen from the same classic and commonly used algorithms, such as TD3 and DDPG algorithms. Figures 6, 7 shows the completion of these two algorithms performing the lane keeping task in the same environment. The first row of them shows the results of TD3 algorithm and the second row shows the results of DDPG algorithm. From the above figure, it can be seen that the traditional TD3 algorithm and the DDPG algorithm, although they can also successfully accomplish the lane keeping task, are not as good as the PER-TD3 algorithm in terms of execution results and algorithm performance.

In the following, we make a detailed comparative analysis of the several algorithms from different perspectives. In order to evaluate the effect of our experiment more visually, we show it on a graph.

**Figure 8:**
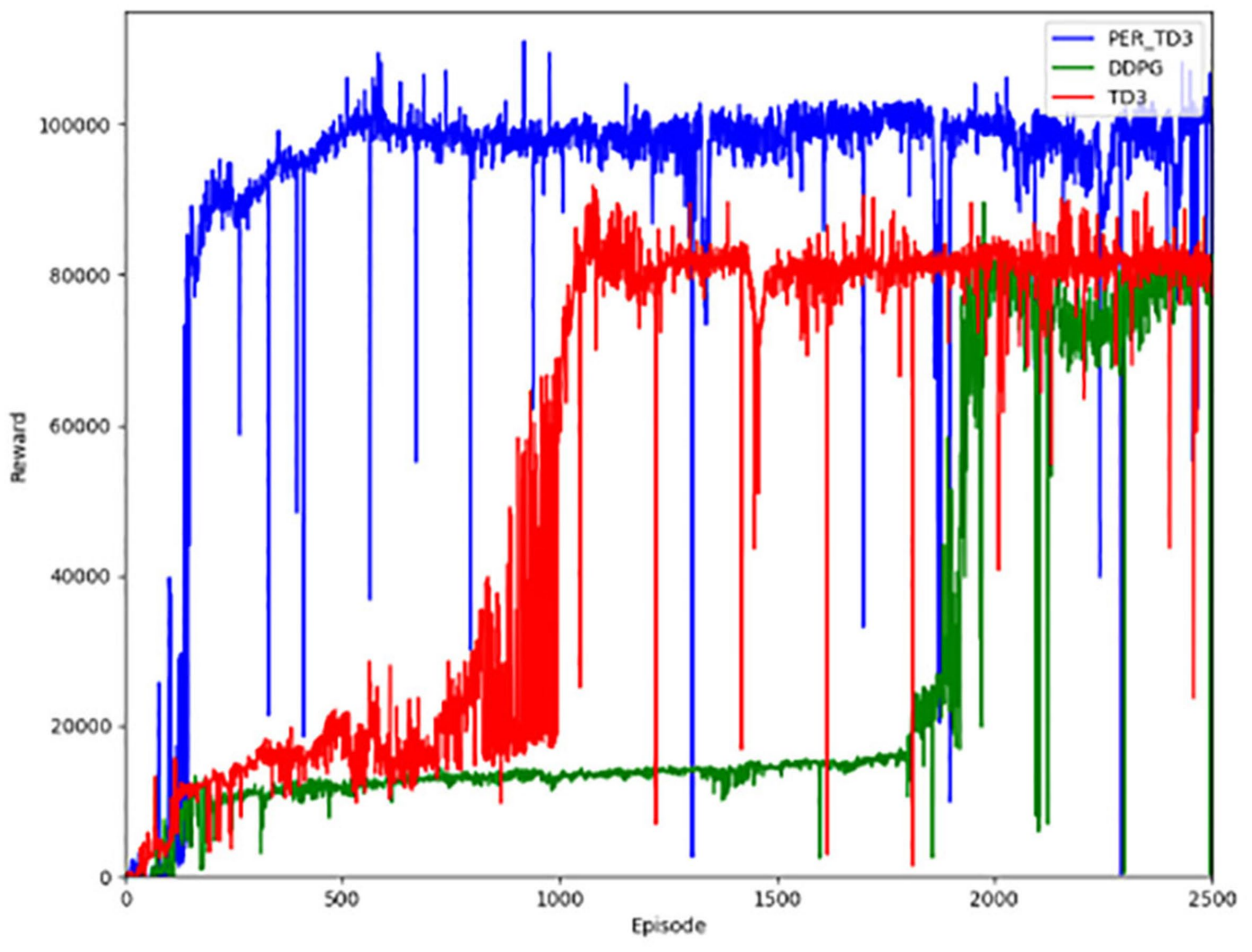
Reward values for the baseline.

**Figure 9:**
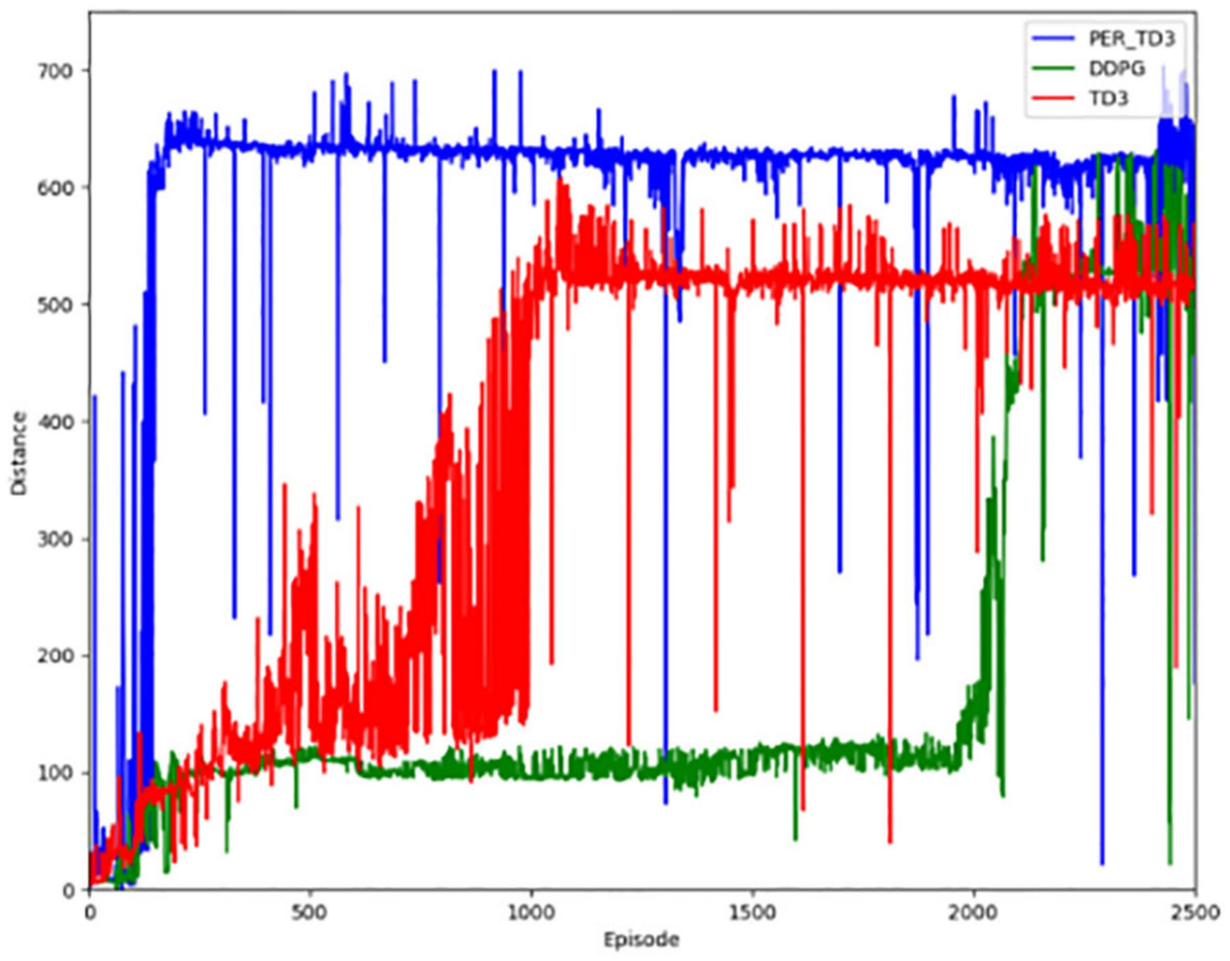
Safe driving distance for the baseline.

The first contrast is in terms of the reward function. As shown in Figure 8, the PER-TD3 algorithm has improved in terms of reward value as a result of the incorporation of the prioritized playback mechanism. In addition, it is able to converge faster than the other two algorithms, as can be seen in Figure 8 presents the cumulative reward learning curves over 500 training episodes. The PER-TD3 algorithm stabilizes at a relatively high level, while TD3 requires more episodes to converge and achieves slightly lower performance. DDPG exhibits a larger performance gap due to the persistent overestimation problem. These trends are consistent across multiple independent runs with different random seeds, demonstrating the reliability of the results. Figure 9 compares the safe driving distances, which are directly related to the reward function. Across repeated experiments, the PER-TD3 algorithm consistently achieves significantly longer safe driving distances without collisions compared to TD3 and DDPG, confirming its superior performance in maintaining safety.

**Figure 10:**
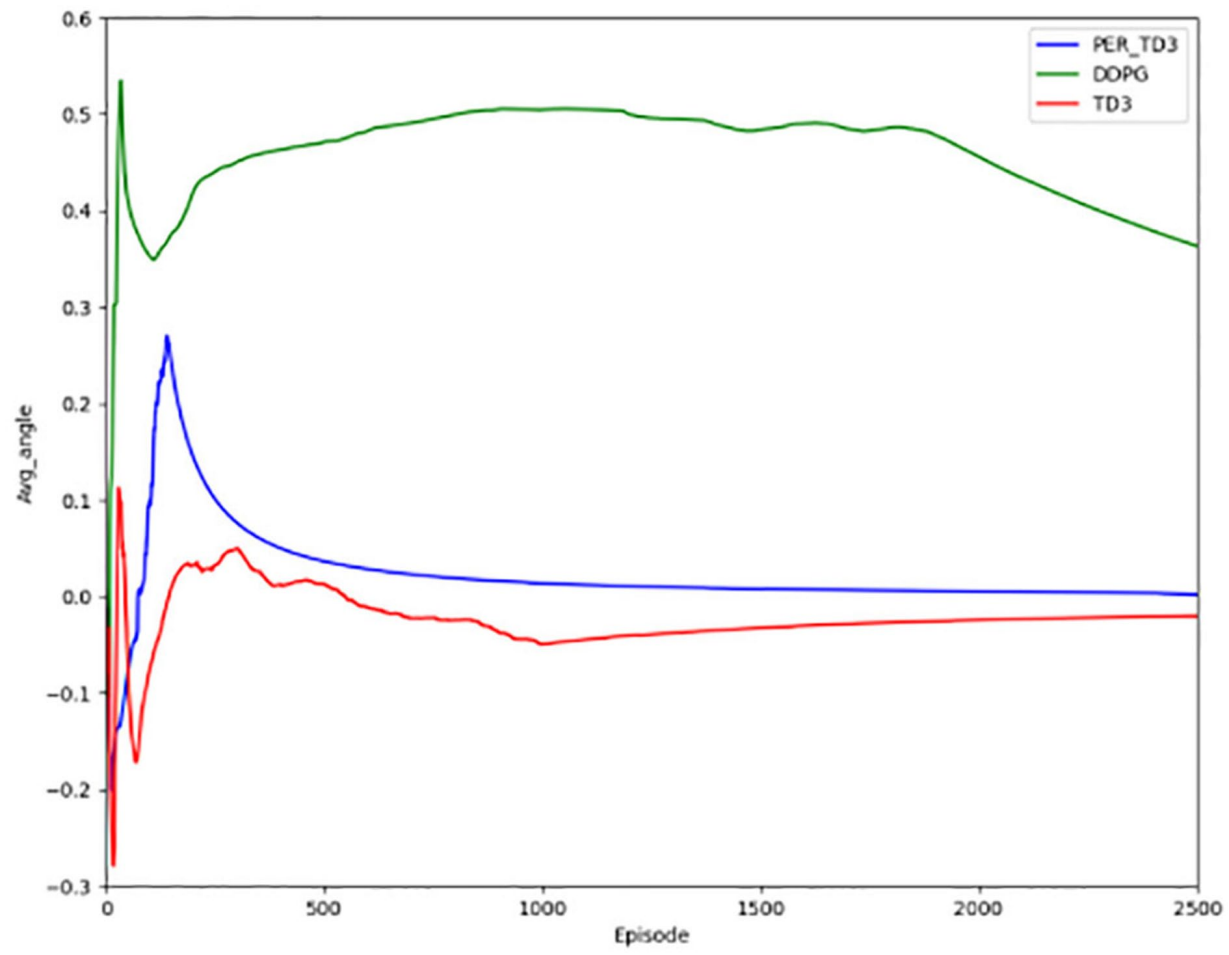
Angle of deviation for the baseline.

**Figure 11:**
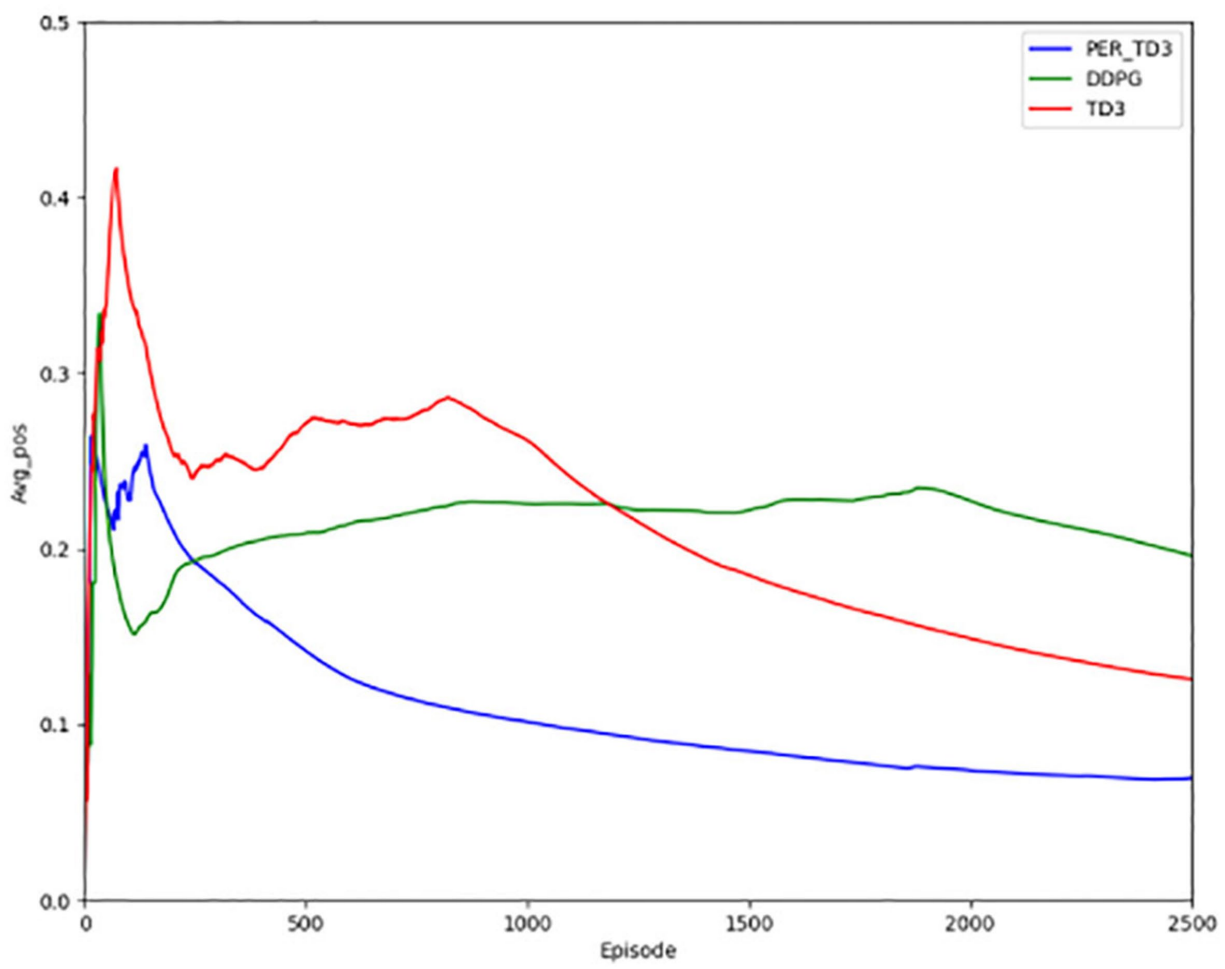
Lateral distance for the baseline.

Figure 10 illustrates steering angle control, where smaller absolute deviations from the road center axis correspond to higher safety. After convergence, the PER-TD3 algorithm maintains steering angles close to zero, indicating precise lane keeping. TD3 performs moderately well in this indicator, while DDPG exhibits larger deviations and requires more training episodes to converge. These patterns remain stable across multiple runs, demonstrating the robustness of the proposed method. Figure 11 Shows the absolute distances between the vehicle and the yellow lane boundaries (positive left, negative right). The PER-TD3 distances converge between 0 and 0.1, outperforming TD3 and DDPG, whose distances remain above 0.1. This consistent behavior across multiple training runs further highlights the stability and generalizability of the PER-TD3 approach.

Overall, the experimental results demonstrate that the PER-TD3 method consistently outperforms baseline algorithms in terms of cumulative reward, safe driving distance, steering precision, and lane boundary control. The trends observed across multiple independent runs indicate both the reliability and generalization capability of the proposed approach.

## 5 Discussion

In this paper, a novel driving task framework PER-TD3 incorporating sample optimization is proposed to specifically solve the lane keeping problem in autonomous driving. Based on the traditional TD3 algorithm, by introducing Prioritized Experience Replay (PER), this framework significantly improves the utilization of high-quality samples and optimizes the algorithm’s performance. The faster convergence is mainly attributed to the prioritized sampling mechanism, which provides better gradient signals by focusing updates on high-TD-error transitions, while the adaptive sampling mechanism reduces variance across different training stages, thereby enabling more efficient accomplishment of the autonomous driving task. Meanwhile, this paper also compares the new framework with the existing mainstream TD3 algorithm and DDPG algorithm. The experimental results show that PER-TD3 shows significant improvement in several key performance indicators, such as reward value, safe driving distance, deflection angle, and the distance between the vehicle and the yellow line at the road edge, thus verifying the effectiveness of the algorithm and ensuring the safety of autonomous driving vehicles. In our future research work, we are also committed to integrating the latest improvement techniques of DQN into the PER-TD3 framework to enhance the algorithm’s decision-making and adaptability in dealing with complex environments as well as to develop a multitask learning strategy, which enables autonomous driving vehicles to simultaneously learn tasks such as overtaking and lane changing, on-ramp merging, and emergency obstacle avoidance, thus enhancing the framework’s versatility and practicality. Finally, we intend to investigate methods for transferring the learned policies from simulation to real-world driving scenarios, including domain adaptation and transfer learning techniques, to bridge the gap between simulated and real-world environments.

## Data Availability

The raw data supporting the conclusions of this article will be made available by the authors, without undue reservation.
